# Creation of Functional Viruses from Non-Functional cDNA Clones Obtained from an RNA Virus Population by the Use of Ancestral Reconstruction

**DOI:** 10.1371/journal.pone.0140912

**Published:** 2015-10-20

**Authors:** Ulrik Fahnøe, Anders Gorm Pedersen, Carolin Dräger, Richard J Orton, Sandra Blome, Dirk Höper, Martin Beer, Thomas Bruun Rasmussen

**Affiliations:** 1 DTU National Veterinary Institute, Technical University of Denmark, Lindholm, Kalvehave, Denmark; 2 Center for Biological Sequence Analysis, DTU Systems Biology, Technical University of Denmark, Kgs. Lyngby, Denmark; 3 Institute of Diagnostic Virology, Friedrich-Loeffler-Institut, Greifswald-Insel Riems, Germany; 4 Institute of Biodiversity, Animal Health, and Comparative Medicine, College of Medical, Veterinary and Life Sciences, University of Glasgow, Glasgow, United Kingdom; 5 MRC–University of Glasgow Centre for Virus Research, Institute of Infection, Inflammation and Immunity, College of Medical, Veterinary and Life Sciences, University of Glasgow, Glasgow, United Kingdom; University of California Davis, UNITED STATES

## Abstract

RNA viruses have the highest known mutation rates. Consequently it is likely that a high proportion of individual RNA virus genomes, isolated from an infected host, will contain lethal mutations and be non-functional. This is problematic if the aim is to clone and investigate high-fitness, functional cDNAs and may also pose problems for sequence-based analysis of viral evolution. To address these challenges we have performed a study of the evolution of classical swine fever virus (CSFV) using deep sequencing and analysis of 84 full-length cDNA clones, each representing individual genomes from a moderately virulent isolate. In addition to here being used as a model for RNA viruses generally, CSFV has high socioeconomic importance and remains a threat to animal welfare and pig production. We find that the majority of the investigated genomes are non-functional and only 12% produced infectious RNA transcripts. Full length sequencing of cDNA clones and deep sequencing of the parental population identified substitutions important for the observed phenotypes. The investigated cDNA clones were furthermore used as the basis for inferring the sequence of functional viruses. Since each unique clone must necessarily be the descendant of a functional ancestor, we hypothesized that it should be possible to produce functional clones by reconstructing ancestral sequences. To test this we used phylogenetic methods to infer two ancestral sequences, which were then reconstructed as cDNA clones. Viruses rescued from the reconstructed cDNAs were tested in cell culture and pigs. Both reconstructed ancestral genomes proved functional, and displayed distinct phenotypes *in vitro* and *in vivo*. We suggest that reconstruction of ancestral viruses is a useful tool for experimental and computational investigations of virulence and viral evolution. Importantly, ancestral reconstruction can be done even on the basis of a set of sequences that all correspond to non-functional variants.

## Introduction

Positive-strand RNA viruses, including classical swine fever virus (CSFV), have high mutation rates compared to eukaryotic organisms due to the lack of proofreading by their RNA-dependent RNA polymerase [1]. This inherently high mutation rate results in extensive genetic heterogeneity of virus populations with viral variants that are phenotypically distinct [2]. The genetic heterogeneity is beneficial to the virus populations in the sense that it enables rapid adaptation and escape from host antiviral responses, but it is also potentially harmful because many mutations may be detrimental or even lethal for the viruses. Indeed, several studies have revealed a high proportion of deleterious mutations leading to lethal or low-fitness phenotypes [3], [4]. The fidelity of the RNA-dependent RNA polymerase has been shown to affect virulence with increased fidelity leading to attenuation and restricted diversity in the viral population [5], [6]. Generally, a high mutation rate will cause a population to consist of a swarm of different, but closely related, genotypes forming a flat fitness landscape that makes the population more robust to mutations [7]. It has been proposed that viral populations with a high diversity of genotypes will have a better chance of surviving host responses to infection [8]. For CSFV, high virulence has been related to high diversity [9].

The analysis of viral genotype composition has typically relied on clonal approaches and subsequent sequencing of partial genomes. However, Next-Generation Sequencing (NGS) has rapidly become the preferred technology for addressing this issue [10]. Using NGS it is possible to sequence a large number of individuals in a population of viral genomes at potentially great depths. This allows for detection of single nucleotide variants (SNV) present at low frequencies within a virus population. However, the linkage of SNVs in separate parts of the genome is not easily discerned from SNV analysis of NGS data because of read length limitations. Attempts have been made to overcome sequencing errors introduced by NGS technology and use the data to model the fitness landscape of a virus population [11] and for full-length haplotype reconstruction [12]. However, all described NGS approaches have limitations concerning the assessment of the functionality of each individual haplotype.

Here we present a study of the evolution in the CSFV strain “Roesrath”, a recent European genotype 2.3, which displays moderate virulence in domestic pigs and wild boar [13], [14]. This was done using a combination of deep sequencing and analysis of multiple, individual, full-length cloned cDNAs. This is, to our knowledge, the first study that combines deep sequencing and functional analysis of full-length cloned cDNAs representing the spectrum of individual genotypes to investigate the evolution of a viral population. We find that the majority of investigated clones (82%) contain lethal mutations. Based on the isolated clones (most of which are non-functional), we reconstruct two ancestral forms of the virus, which we demonstrate to be functional using *in vitro* and *in vivo* analyses.

## Materials and Methods

### Virus isolates

The CSFV strain “Roesrath” was used for the experiments (CSFV/2.3/wb/ CSF1045/2009/Roesrath; Genbank accession number GU233734). Two different cell culture passages derived from the same isolate were used: the CSFV_Roesrath_P5, which was a fifth passage sample from PK-15 cells whereas the CSFV_Roesrath_P2 was a second passage sample of the same isolate.

### Generation of cloned cDNAs

The cloned cDNAs were produced from CSFV RNA as described previously [15], [16], [17]. Briefly, viral RNA was extracted from CSFV_Roesrath_P5 by a combined Trizol/RNeasy protocol. Subsequently, the viral genomes were amplified by RT-PCR to generate full-length genome amplicons flanked by *Not*I sites and with a T7 promoter upstream of the cDNA sequence using primers CSFV-Ros_Not1-T7-1-59 and CSFV-Ros_12313aR_Not1 (Table 1). Fragment termini were digested with *Not*I and products were ligated into the bacterial artificial chromosome (pBeloBAC11). Individual colonies were propagated on LB plates supplemented with chloramphenicol, and screened following restriction enzyme digestion. PCR products for the *in vitro* transcription and the sequencing were obtained from each cloned cDNA using same forward primer and CSFV-Ros_12313aR (Table 1).

**Table 1:** Primers used in this study.

Primer	Sequence 5′ – 3′
CSFV-Ros_Not1-T7-1-59	5′-TCA TAT GCG GCC GCT AAT ACG ACT CAC TAT AGT ATA CGA GGT TAG CTC GTT CTC GTA TAC GAT ATC GGA TAC ACT AAA TTT CG-3′
CSFV-Ros_12313aR_Not1	5′-ATA TGC GGC CGC GGG CCG TTA GGA AAT TAC CTT AGT CCA ACT GT-3′
CSFV-Ros_12313aR	5′-GGG CCG TTA GGA AAT TAC CTT AGT CCA ACT GT-3′
CSFV-Ros_cDNA	5′-GGGCCGTTAGGAAATTACCTTAGT-3′
CSFV-Ros-8093-F	5′-GGAGCTGTAGCAGCCCACAATGC-3′
CSFV-Ros-4018-F	5′-GACTTGGCTACAGTACCTCGTCAGC-3′
CSFV-Ros-4599-R	5′-GAAACGAGGTTGGTCCCACCAGC-3′
CSFV-Ros-1180-F	5′-CCAGCCCGTGGCAGCCGAGAAC-3′
CSFV-Ros-2107-R	5′-CAGGTTCTTCGTGGGACTGGGGG-3′
CSFV-Ros-8512-F	5′-CAATCAGCTGGGCCCCCGCC-3′
CSFV-Ros-9435-R	5′-CCCCAGTATCAGTACCGAGGGCC-3′

### Testing of RNA transcripts from full-length cDNA clones *in vitro*

cDNAs were transcribed using a Megascript T7 RNA transcription kit. Run-off RNA transcripts were electroporated into porcine PK-15 cells and incubated at 37°C in Eagles medium with 5% FCS. After 72 hours plates were stained for the presence of pestivirus antigens using biotinylated pig anti-CSFV/BVDV polyclonal IgG followed by avidin-conjugated horseradish peroxidase (eBioscience) for detection of viral proteins using microscopy. Cell supernatants from the replication competent transcripts were passaged onto uninfected PK-15 cells and incubated for a further 72 hours.

### Full length sequencing of cloned cDNAs

Full-length sequences of the PCR products obtained from each cloned cDNA were determined using Ion Plus fragment library kit (Life technologies) and sequenced by the Ion PGM platform (Life technologies) or by using a Miseq instrument (Illumina) with libraries constructed by the SPRI-TE library system (Beckman Coulter) using Illumina indices on a SPRIworks Fragment Library Cartridge II (Beckman Coulter). Sequencing data were assembled by the Newbler *de novo* assembler (Roche) and mapped to the CSFV Roesrath reference sequence (GU233734) by the BWA aligner using the BWASW algorithm [18] and processed by Samtools [19]. Consensus sequences of all clones were aligned using the MAFFT algorithm in Geneious R7.

### Deep sequencing of parental virus sample

The RT-PCR products obtained from the original CSFV_Roesrath_P5 sample (which was used to generate the cDNA clones) were deep sequenced with both the FLX genome sequencer (Roche) using the SPRIworks Fragment Library System II (Beckman Coulter, Krefeld, Germany) and the Ion PGM platform using the Ion Plus fragment library kit (Life technologies). The FLX and Ion PGM data was corrected for homo-polymer errors by the RC454 tool using 454 settings [20]. This tool integrates the Mosaic aligner [21] for mapping the reads to the reference sequence. Samtools were applied for bam file processing and SNVs were called by V-Phaser2 and Lofreq for comparison [22], [23]. Subsequently, the SnpEffect tool was used to assess SNV effects [24]. We found very good agreement in SNV distributions between the Ion PGM and the FLX SNV indicating that the SNV calls were reproducible and were not biased much by individual sequencing platforms.

### Phylogenetic analysis and ancestral reconstruction of internal nodes

cDNA clone sequences aligned by MAFFT were compared to the Roesrath reference sequence and mutations were categorised as silent, missense, deletions or situated within the 5′ UTR or 3′ UTR using Geneious R7. Student's t-tests were used to compare SNV frequencies in Graphad Prism 6.0.e. The alignment was analysed using jModelTest 2.1.5, which found the general time reversible model (GTR) to be most suitable substitution model. Phylogeny was reconstructed using MrBayes v3.2.1 [25], [26] on a full-length cDNA sequence alignment (GTR, nst = 6). The Markov chain Monte Carlo algorithm was run for 20,000,000 iterations, with a sampling frequency of 14400, using two independent runs with three chains each in order to check for convergence. Burn-in was set at 25% of samples. The consensus tree was visualized in FigTree v.1.4.0.

Ancestral reconstruction of the internal nodes was performed using PAML [27]. The BaseML program was applied on the full-length nucleotide alignment using GTR as substitution model. The internal node sequences were aligned by MAFFT in Geneious R7.

### Reconstruction of haplotypes by site-directed mutagenesis

The reconstruction of cDNA clones was performed as previously described [15]. Briefly, the megaprimer was generated using CSFV-Ros-1180-F and CSFV-Ros-2107-R as primers and Ros16B as template. The purified PCR product was used as megaprimer for megaPCR with Ros35C as template. Second round PCR was performed with CSFV-Ros-8093-F and CSFV-Ros-9435-R as primers and Ros16B as template for the megaprimer. Subsequently, Ros35C.2 was used as template for the megaPCR generating Ros35C.2.1, which was renamed Ros being a 100% match to the consensus sequence and the deepest node in the phylogeny. Two rounds of site-directed mutagenesis also generated Ros_S1359N_A2668T that corresponds to the node of the largest monophyletic subgroup. Initially, primer CSFV-Ros-4018-F and CSFV-Ros-4599-R were used together with Ros9C as template for the megaprimer. Ros was used as template together with the megaprimer for the MegaPCR that generated Ros_S1359N. The last round megaprimer was obtained with CSFV-Ros-8093-F and CSFV-Ros-9435-R as primers and Ros9C as template, which was used along with Ros_S1359N as template to produce Ros_S1359N_A2668T. This clone was shown by full-length sequencing to only include the two missense mutations (S1359N and A2668T) compared to Ros. Sequences for individual cDNA clones (e.g. Ros9C, Ros16B, Ros35C, Ros35C.2, Ros35C.2.1) can be obtained upon request.

### Testing of virus rescued from reconstructed cDNAs *in vitro*

Virus growth was determined by growth curves as previously described [28]. Briefly, rescued virus was used to infect PK-15 cells (multiplicity of infection 0.1 TCID50/cell) and cultured for 3 days. At 3, 12, 24, 48 and 72 hours total RNA was isolated after a freeze/thaw cycle of cells with medium, and the CSFV genome copy numbers then measured by RT-qPCR [29]. The viral replication assay was adapted from Tamura et al. [30]. Briefly, 300,000 PK-15 cells were infected at a multiplicity of infection of 1.5 TCID50/cell, RNA from cells was harvested at 2, 8 and 12 hours, and the level of viral RNA then measured as described above.

### Testing of rescued viruses *in vivo*

Fifteen weaner pigs were divided into 3 groups of 5 pigs that each received a different inoculum (Group 1: vRos; Group 2: CSFV_Roesrath_P2; Group 3: vRos_S1359N_A2668T). The pigs were inoculated using a vaporizer device (2 ml oral and nasal) with 10^5^ TCID_50_/100 μl. Body temperature and clinical signs were observed on a daily basis. Blood samples and oral swabs were taken at days 0, 2, 4, 7, 10, 14, 21, 28 and 42 post-infection. After day 42, the pigs were sacrificed (electro-stunning and subsequent exsanguination) and subjected to post-mortem examination and sampling. The IDEXX CSFV antibody ELISA (IDEXX, Bern, Switzerland) and neutralisation tests were performed on the serum samples as previously described [31]. Viral RNA was detected using CSFV RT-qPCR protocols for both blood and oral swab samples as described [29].

### Ethics statement

In the framework of the reported animal trial, all applicable animal welfare regulations, including EU Directive 2010/63/EC and institutional guidelines, were taken into consideration. The animal experiment was approved by the competent authority (Landesamt für Landwirtschaft, Lebensmittelsicherheit und Fischerei Mecklenburg-Vorpommern, Germany) under reference number 7221.3–2.5-012/13.

## Results

### Assessment of fitness distribution in the virus population

**Fig 1:**
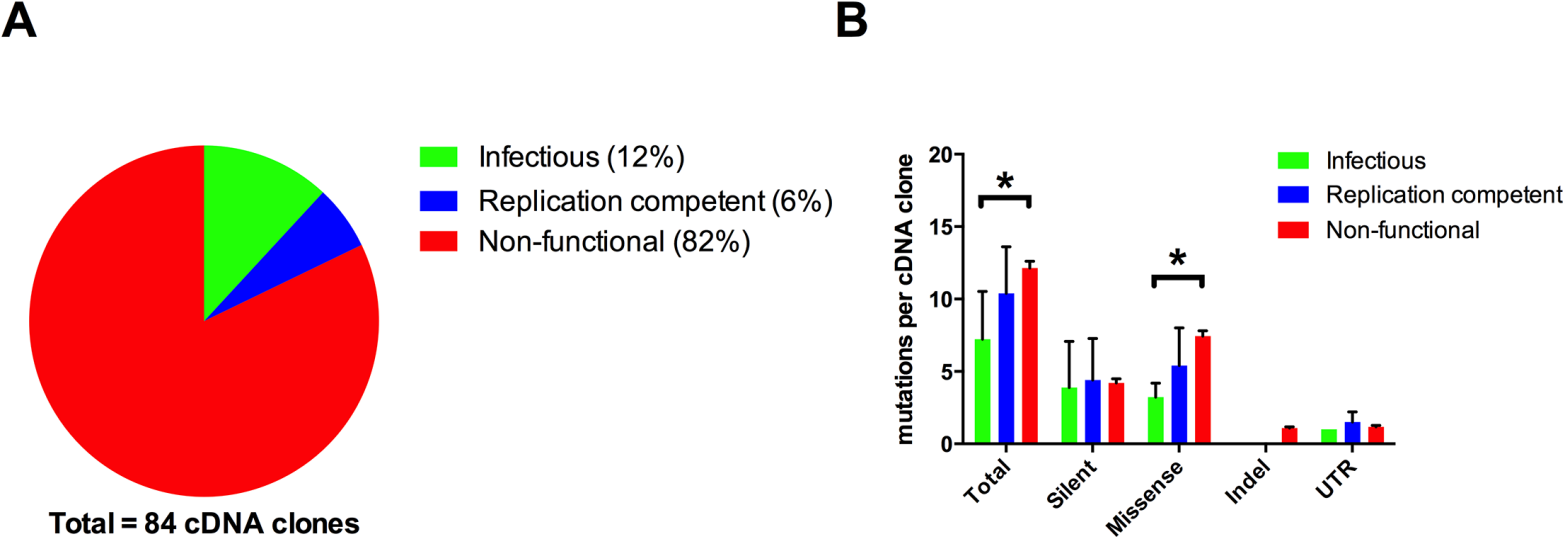
Phenotypic and mutational distribution of the cDNA clones. A) Fraction of cDNA clones found to be infectious, replication competent, or non-functional. B) Number of mutations of different types (silent, missense, insertion/deletion and untranslated region), in the three functional cDNA classes (infectious, replication competent, and non-functional). The average number per cDNA clone is shown; error bars depict standard deviations. Non-functional cDNAs were found to have significantly more missense mutations than infectious cDNAs (p = 3.3E-5, Student's t-test). They also displayed significantly more mutations in total (p = 0.0002).

Eighty-four unique complete cDNAs directly cloned into bacterial artificial chromosome (BAC) vectors were generated from full-length RT-PCR products obtained using RNA extracted from a fifth passage of the CSFV “Roesrath” isolate (termed CSFV_Roesrath_P5). RNA transcripts were produced from individual cloned cDNAs and were tested for replication competence in PK-15 cells. Transcripts from 15 of the cDNA clones (18%) were scored as functional and replicated in PK-15 cells whereas 69 cDNAs (82%) were non-functional without any indication of RNA replication (Fig 1A). For the 15 replicating cDNA clones differences in replication efficiency were observed with varying phenotypes ranging from all cells producing viral proteins to only a few small foci of infected cells. In order to address these differences, harvests from cells displaying CSFV protein production following introduction of the viral RNA transcripts were passaged once on PK-15 cells to establish whether infectious virus had been produced. Ten cDNA clones (12%) were identified as producing virus progeny after this additional passage and were classified as “infectious” whereas the cDNA clones yielding non-infectious RNA transcripts, although producing detectable viral protein, were termed “replication competent” (Fig 1A).

### Clonal sequencing, distribution of mutations, and correlation with phenotype

The full-length sequences of a subset (70 cDNA clones) of the 84 cDNA clones were determined in order to identify individual genotypes within the viral population. This included all of the infectious and replicating cDNA clones as well as the majority (55 out of 69) of the non-functional cDNA clones. The sequence of each individual cDNA clone was determined from *de novo* assembly of sequencing reads. All cDNA clones were found to have unique sequences and had on average 11.4 substitutions out of 12297 nt, compared to the CSFV “Roesrath” reference sequence (GU233734). Out of all observed SNVs in the coding sequence, 38% were silent mutations while 62% were missense mutations (resulting in amino acid changes). This distribution is close to what would be expected for random mutations with no subsequent selection: due to the structure of the genetic code, about two-thirds of random mutations will cause amino acid changes. This observation is consistent with the idea that the circulating virus particles essentially represent the untested offspring of the previous generation of viruses. Frame-shift causing indels (with lengths of 1–2 nt) were observed for 12 out of the 55 non-functional cDNA clones. It was found that a significantly higher number of missense mutations were present in non-functional cDNA clones compared to those that yielded infectious RNAs (t-test, p = 3.3E-5), whereas the number of silent mutations per clone was approximately the same in the two classes. The number of missense mutations in the replication competent (but non-infectious) cDNA clones was between that of the infectious and non-functional clones. This distribution of mutations indicates an association between the number of missense mutations and a decrease in functionality, suggesting that most amino acid changes are not adaptive, in accordance with previous work on the distribution of fitness effects in RNA viruses [3], [4], [32]. Further analysis revealed that only the NS5B protein (RNA-dependent RNA polymerase) had significantly less missense mutations in the infectious compared to the non-functional cDNA clones (t-test, p = 0.003), while the remaining missense mutations were randomly dispersed along the genome. This suggests that most changes in the RNA-dependent RNA polymerase have important fitness effects.

### Phylogenetic structure of the viral population

**Fig 2:**
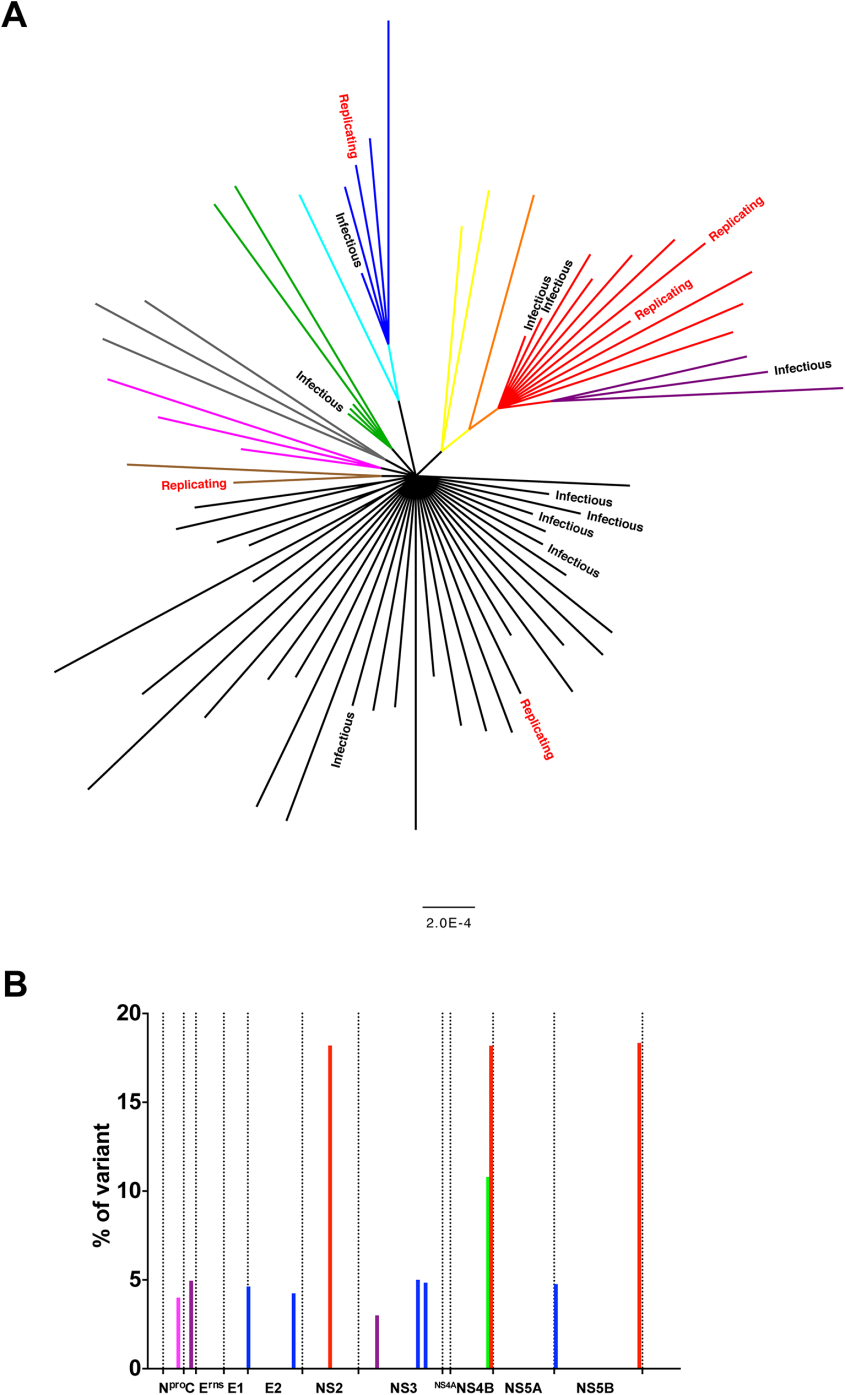
Phylogenetic structure of cDNA clones. A) Phylogenetic structure of the cDNA clone population (tree reconstructed using MrBayes). All viable clones are labelled as either infectious (fully functional) or replicating (capable of replicating in PK-15 cells, but do not produce infectious viral particles). Unlabelled leafs were non-functional. Different haplotypes are shown as coloured clades. B) Haplotype mutation frequency distribution. The plot shows the frequency of SNVs that define the major haplotypes from the NGS deep sequencing. The colour coding corresponds to the respective haplotypes in Fig 2.

In order to further understand the population structure, the phylogenetic tree for the cDNA clones was inferred using Bayesian methods (Fig 2A). The unrooted tree structure was mostly star-like, with a majority of cDNA sequences branching out from a single deep ancestral node, which represents the Roesrath reference sequence, along with additional monophyletic groups marked in colour on Fig 2A. Labeling individual leafs (representing individual cDNA clones) according to functionality, showed that sequences belonging to the infectious and replication competent variants were distributed over most of the subgroups and that functional variants were present in all of the major groups. The leaves for the infectious variants were found to be closer to the internal nodes than those that were non-functional, in agreement with the mutation distribution (Fig 1B). For three of the monophyletic subgroups, more than one mutation was observed (making those mutations linked with one another on the viral genome for that group) with two groups having five mutations (purple and blue) and one having three (red). For example, the red subgroup has three linked mutations: two missense mutations, one each in NS2 and NS4B, and a silent mutation in NS5B.

### Deep sequencing analysis of SNVs in the parental virus population

The cDNA that was obtained from CSFV_Roesrath_P5 and used as input for the cloning procedure was deep sequenced to investigate the SNV distribution within the parental un-cloned viral population. An independent, full-length RT-PCR product obtained from the same CSFV_Roesrath_P5 sample was also deep sequenced. Table 2 shows the SNV distribution from these data, after error correction and filtering. All observed SNVs displayed frequencies below 20% and the consensus sequences generated from both data sets were identical to the Roesrath reference sequence. Nearly all mutations present in more than one of the cloned cDNAs, could also be detected in the SNV data. Unique mutations in the cDNA clones might be caused by last round replication errors, or they may be low frequency SNVs in the populations (<1%). Subsequently, the focus was put on mutations appearing in more than one cDNA clone. Individual SNVs (Table 1) were colour-coded to match the branch colours used in the phylogeny (Fig 2A). Three groups of haplotypes with more than one SNV could be recognized in the tree. These three groups are coloured blue, red and purple (the latter being a subclade of the red group). SNVs within the purple and blue haplotype groups had very similar frequencies (about 5%) within the population (Fig 2B). It would therefore not be possible to determine whether SNVs in these two groups were linked or not merely by looking at their frequencies alone, and only in combination with the phylogeny generated from the full-length cDNA clone sequences could these genotypes be distinguished.

**Table 2:** SNV analysis of the viral population (CSFV_Roesrath_P5) by NGS deep sequencing.

Protein	Nucleotide position	Roesrath reference (GU233734)	Variant	SNV Total (%) ^a^	SNV FLX (%) ^a^	SNV PGM (%) ^a^	Variant (aa)	Haplotype in cDNA clones ^b^

**N** ^**pro**^	787	T	C	1.1	0.9	1.2	Silent	Present
**C**	919	C	T	1.2	0.9	1.3	Silent	Not found
	938	C	T	4.2	4.2	4.2	Silent	Present
	1058	A	T	5.0	5.1	4.9	**T229S**	P
**E** ^**rns**^	1673	G	A	4.1	4.5	3.9	**G434R**	Present
	1750	C	T	2.2	2.7	2	Silent	Not found
**E2**	2459	G	A	4.6	3.8	4.8	**D696N**	B
	3559	T	G	4.2	3.5	4.4	Silent	L, B
**p7**	3622	G	A	2.2	2.3	(2.2)	Silent	Not found
	3637	C	T	2.8	3.1	2.8	Silent	Present
**NS2-3**	3847	C	A	2.1	2.3	2	Silent	Present
	4171	A	G	1.8	(1.4)	1.9	Silent	Not found
	4449	G	A	18.2	18.3	18.2	**S1359N**	R, P, Y, O
	5593	C	G	3.5	(3.5)	(5.1)	Silent	P
	5998	T	C	2.7	3.2	2.5	Silent	Not found
	6541	T	C	4.1	4.5	4	Silent	Present
	6592	T	A	5	4.2	5.3	Silent	B
	6781	C	T	4.8	4.4	5	Silent	L, B
**NS4B**	8220	A	G	2.4	(2.1)	2.5	**K2616R**	Not found
	8302	T	C	10.8	9.1	11.2	Silent	G
	8375	G	A	18.2	18.1	18.2	**A2668T**	R, P, O
**NS5B**	9958	T	C	4.8	4.4	4.9	Silent	L, B
	10915	T	C	1.1	(0.7)	1.1	Silent	Present
	11101	A	G	3.4	3	3.5	Silent	Not found
	11407	C	T	3.0	3	3	Silent	Not found
	11533	A	G	2.4	2.3	2.4	Silent	Present
	11572	C	T	2.4	2.4	2.4	Silent	Present
	11575	C	T	3.8	4.2	3.7	Silent	Present
	11992	T	C	18.3	18.2	18.4	Silent	R, P

^a^ Two independent populations of PCR products obtained from the viral population (CSFV_Roesrath_P5) were NGS deep sequenced using the FLX and Ion PGM platforms. SNV frequencies are shown, as percentages, for both datasets combined, called by both Lofreq and V-Phaser 2 (SNV Total), followed by the FLX and Ion PGM SNV percentages respectively. SNVs frequencies called only with V-phaser 2 are shown in parenthesis.

^b^ This column indicates which cDNA clone haplotype the SNV belongs to. Colour codes correspond to haplotypes used in the phylogeny and the mutation frequency distribution in Fig 2A and 2B. P: Purple; B: Blue; L: Light blue; Y: Yellow; O: Orange; R: Red; G: Green.

### Computational reconstruction of internal nodes in the phylogeny of the virus population

**Fig 3:**
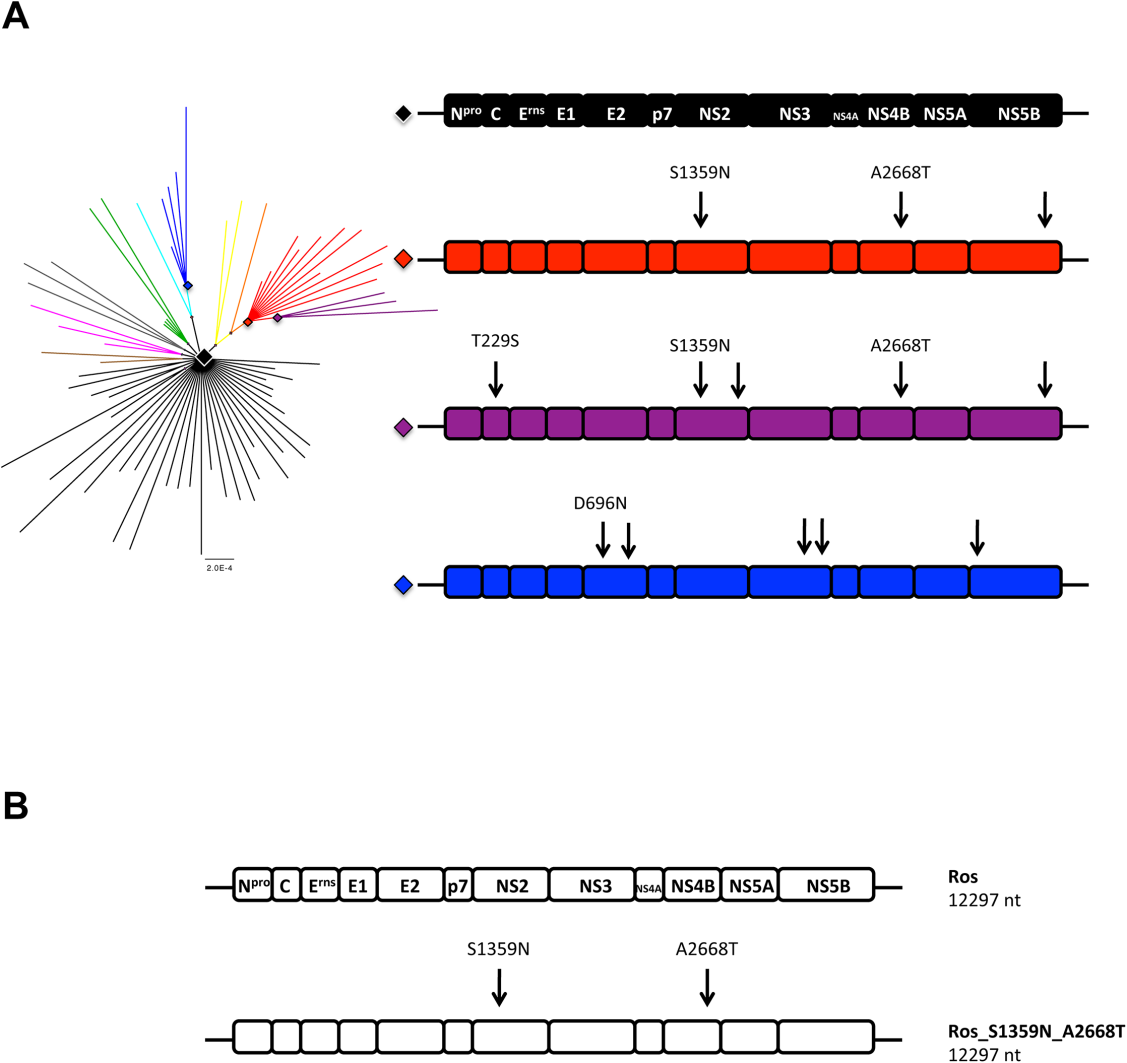
Ancestral reconstruction of major internal nodes. **A)** Structure of computationally inferred ancestral sequences, corresponding to the four internal nodes indicated by diamond shapes. The black node was found to be identical to the Roesrath reference sequence (GU233734). The structures of the predicted ancestral genomes for the red, purple and blue nodes are shown with differences to the black node indicated (arrows). Mutations leading to aa changes are labeled. B) Reconstructed cDNAs corresponding to the two major internal nodes.

As mentioned above, the majority of cloned viral sequences were non-functional. However, the closer a cDNA clone was to an internal node in the phylogeny the more likely it was to be functional, an observation also made by other groups [33]. Since each circulating virus must be the descendant of a functional ancestor, we suggest that ancestral reconstruction of sequences corresponding to internal nodes will typically lead to fully functional and infectious virus variants (the ancestor itself must have been functional, but the inferred ancestral sequence may not always be correct). To test this hypothesis we performed ancestral reconstruction using PAML for sequences corresponding to internal nodes in the phylogeny (Fig 3A). The structure of the predicted ancestral sequences are indicated for the four major groups, and contain both silent and missense mutations compared to the Roesrath reference sequence.

### Analysis of reconstructed ancestral sequences *in vitro*

**Fig 4:**
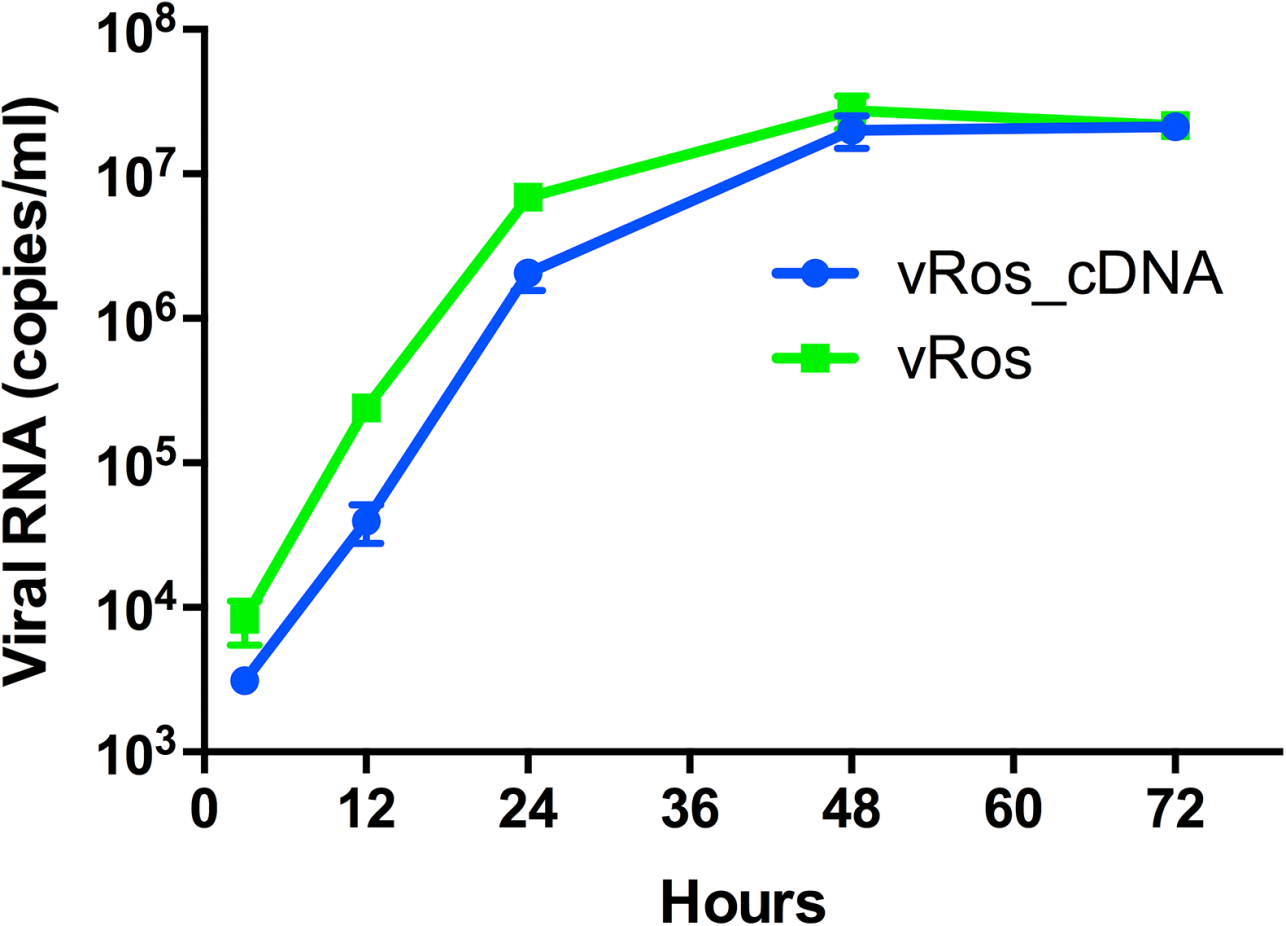
Growth kinetics of the deepest ancestral node. Growth curves of viruses in PK-15 cells were measured using RT-qPCR assays (viral RNA copies/ml) at 3, 12, 24, 48, and 72 hours after infection. Means ± s.d. are shown for biological replicates (n = 3).

**Fig 5:**
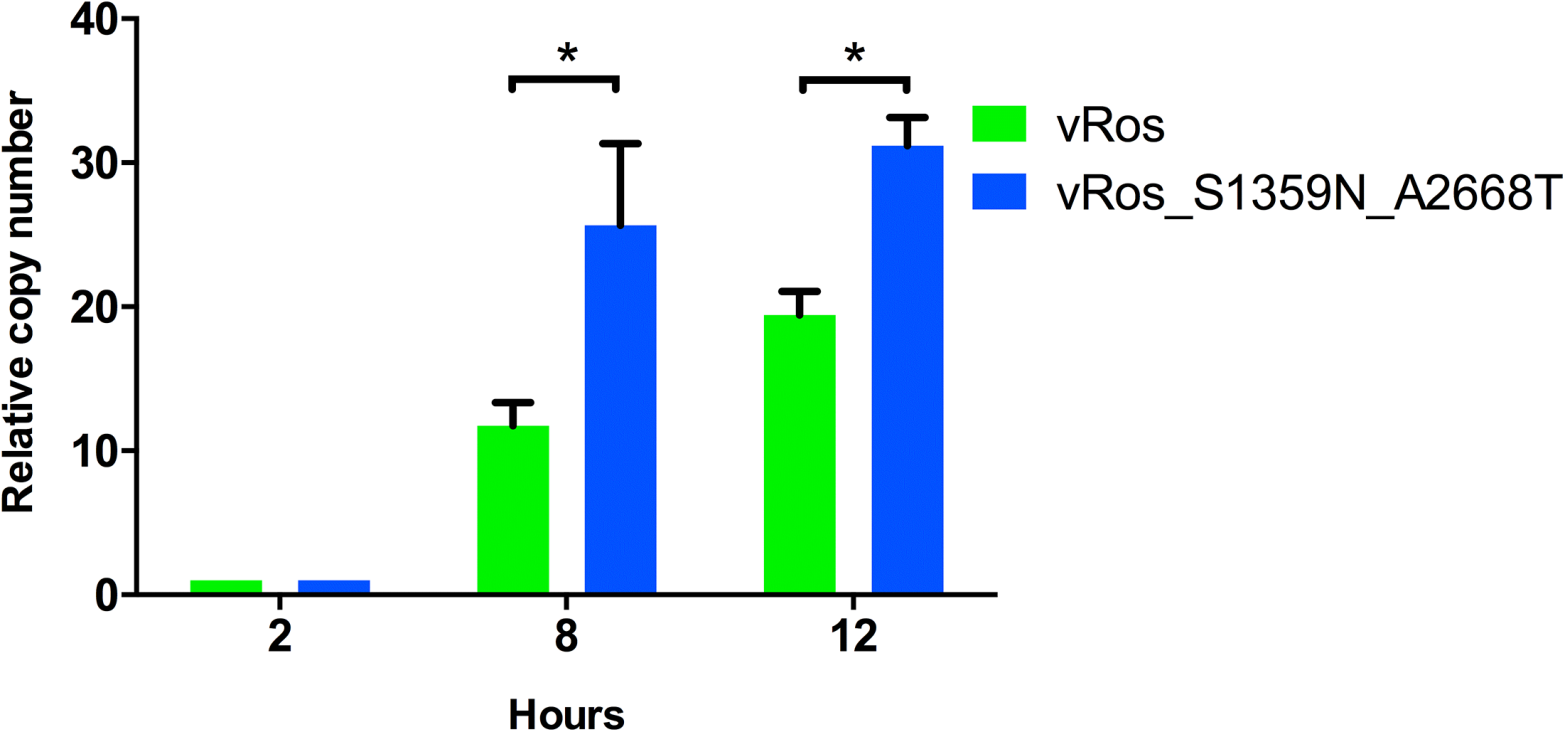
Replication kinetics of both reconstructed ancestral nodes. Replication of viruses in PK-15 cells was measured using RT-qPCR relative to 2 hour measurement at 8 and 12 hours after infection. Means ± s.d. are shown for biological replicates (n = 3). The replication rates of the two constructs was significantly different at both 8h (p = 0.003, t-test) and 12h (p = 0.008).

Based on the computationally inferred ancestral sequences, we produced constructs corresponding to the black and red internal nodes in Fig 3A, with the purpose of testing their functionality *in vitro* and *in vivo*. These constructs were created using site-directed mutagenesis, starting from one of the infectious clones and removing mutations step by step to produce the desired cDNA clones. Specifically, the clone from which we started had 2 missense and one silent mutation compared to the inferred sequence of the black internal node. We first constructed a cDNA clone corresponding to the ancestral sequence at the black diamond shaped node (here termed “Ros”; Fig 3B). This inferred ancestral sequence is identical to the consensus sequence of the viral population. RNA transcripts derived from Ros proved infectious in PK-15 cells, with growth curves showing that the virus rescued from Ros (termed “vRos”) proliferates at least as well as the virus rescued from RNA transcripts obtained from the parental cDNA (vRos_cDNA) in cell culture (Fig 4). Starting from the Ros cDNA clone the ancestral sequence at the red node was subsequently constructed using two additional steps of site-directed mutagenesis. This sequence, named “Ros_S1359N_A2668T”, had the two missense SNVs (S1359N in NS2 and A2668T in NS4B), but not the silent change in NS5B (T11992C; Fig 3B). Each step added one missense mutation and transcripts containing each of the individual changes (Ros_S1359N and Ros_A2668T) were found to be infectious in PK-15 cells (data not shown). The final construct Ros_S1359N_A2668T also proved to be infectious in cell culture. As both ancestral reconstructions led to infectious viruses, we further tested their replication efficiency in cell culture. PK-15 cells were infected with the same infectious dose; RNA was extracted at 2, 8 and 12 hours, and the level of CSFV genomes then measured by RT-qPCR. This analysis showed that the ancestor at the red node, vRos_S1359N_A2668T, replicated significantly faster than the ancestor at the black node, vRos (Fig 5). This was seen at both 8 (t-test, p = 0.003) and 12 hours post infection (t-test, p = 0.008).

The fact that both of these constructs are infectious and replicate well in cell culture, supports one of the main ideas in this work: when working with RNA viruses, ancestral reconstruction may be a useful tool for obtaining functional clones for elucidating molecular determinants of virus function. This can be done even if all sequenced viruses (used as the basis for inferring the ancestral forms) are non-functional.

The observation that vRos virus replicated at a lower rate than vRos_S1359N_A2668T in cell culture suggests that the substitutions S1359N (in NS2) and A2668T (in NS4B) may be adaptations leading to higher replication efficiency of the virus in cell culture. These substitutions have not been described before and both positions are fully conserved in CSFV sequences present in GenBank.

### Deep sequencing of rescued viruses

**Fig 6:**
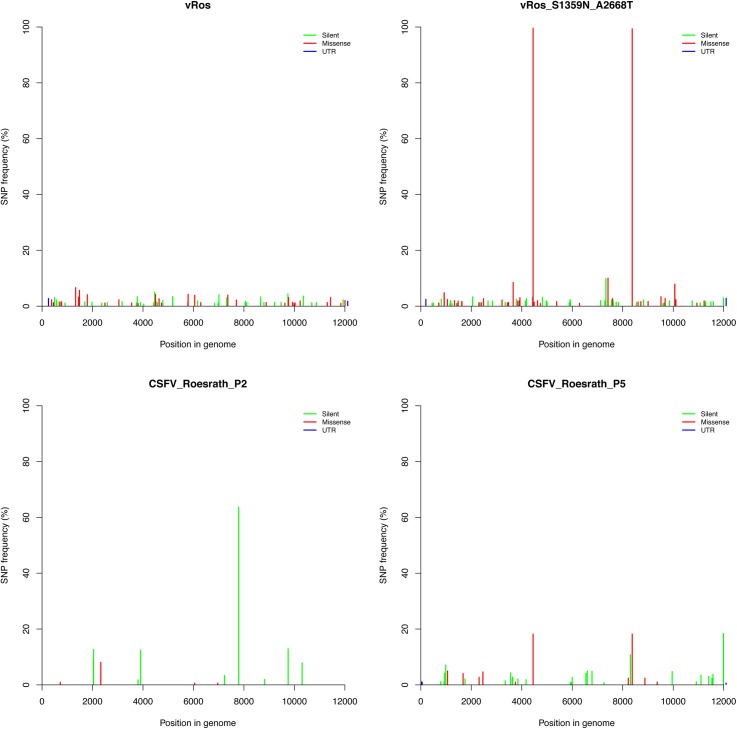
Deep sequencing of virus inoculums for *in vivo* testing. The histograms depict SNV (SNP) frequency on the y-axis and genome position on the x-axis. The blue, green and red colour indicates SNVs grouped as silent, missense or untranslated region (UTR), respectively.

Viruses (vRos and vRos_S1359N_A2668T) rescued from the two reconstructed ancestral cDNAs were deep sequenced and compared to CSFV_Roesrath_P5 (Table 2) used for the initial cloning in order to identify possible substitutions and adaptations to cell culture, A 2^nd^ passage of the same virus isolate (CSFV_Roesrath_P2) was also deep sequenced. Fig 6 depicts the SNV distribution across the cDNAs, mapped to the Roesrath reference sequence. In the vRos and CSFV_Roesrath_P2 sequences only low frequency SNVs could be observed in each population and both had consensus sequences identical to the deepest ancestral node, except for a silent SNV (T7792C) that was present at 64% for the CSFV_Roesrath_P2 sample. This silent SNV was not observed in CSFV_Roesrath_P5 (Table 2). The virus vRos_S1359N_A2668T had a very similar pattern of SNVs as vRos but with the two missense mutations G4449A and G8375A fixed at 100% as expected. The mutations leading to the amino acid (aa) substitutions S1359N and A2668T were observed in the population of CSFV_Roesrath_P5 at about 18% (Table 2) but were not seen (even at low frequency) in CSFV_Roesrath_P2.

### Experimental analysis of reconstructed ancestral sequences *in vivo*

**Fig 7:**
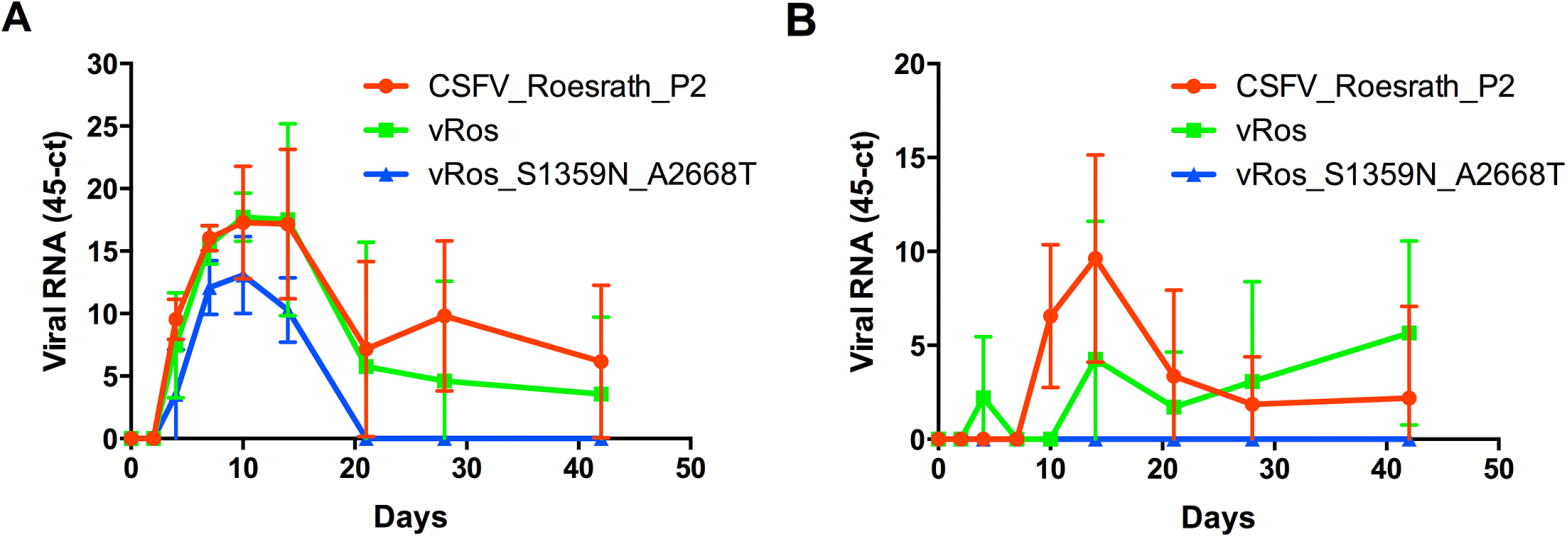
Properties of reconstructed viruses in pigs. A) Level of viral RNA in the blood during the course of infection measured by RT-qPCR (viral RNA copies/ml). B) Level of viral RNA in oral swab samples measured by RT-qPCR (viral RNA copies/ml). Means ± s.d. (n = 5) are shown.

To further investigate the differences in replication rate observed *in vitro* between the two reconstructed ancestral viruses (vRos and vRos_S1359N_A2668T; Fig 5) we also tested the viruses in their natural host. The two rescued viruses were analysed in parallel with the CSFV_Roesrath_P2 to compare their virulence. Three groups of weaner pigs, each with five animals, were inoculated with vRos, CSFV_Roesrath_P2 and vRos_S1359N_A2668T respectively. Mild clinical symptoms were observed in the groups infected with vRos and CSFV_Roesrath_P2 and there were growth impediments for a few of the pigs in each group. Two pigs were euthanized at day 12 post infection from the group inoculated with vRos due to persistent clinical symptoms and declining general health. No disease symptoms were observed for the group inoculated with vRos_S1359N_A2668T. It should be noted that the CSFV strain “Roesrath” is moderately virulent and has been shown to cause highly variable clinical pictures, from subclinical [34], to acute-lethal and chronic [14]. All three groups seroconverted against CSFV by day 28 of the experiment, and anti-CSFV antibodies could be detected as early as day 14. All pigs were scored as positive in a specific neutralisation test after day 14 (data not shown). Viremia was monitored using RT-qPCR assays to measure viral RNA in blood samples taken during the course of the experiment. The groups inoculated with vRos and CSFV_Roesrath_P2 had an almost identical profile of viremia except on day 14 where a higher load of vRos was apparent in the bloodstream (Fig 7A). For both groups viremia declined after day 14, but was detectable at low levels until the end of the experiment on day 42. However, the group infected with vRos_S1359N_A2668T had much lower levels of viral RNA in the blood, with viremia peaking between day 7 and 10 and virus being undetectable after day 14. Indeed, lower levels of viremia were observed for the group infected with vRos_S1359N_A2668T compared to groups inoculated with vRos and CSFV_Roesrath_P2 at every sample time examined. Oral swabs were taken on the same days as blood samples and assayed for viral RNA in the oral cavity using RT-qPCR (Fig 7B). Only the groups infected with CSFV_Roesrath_P2 and vRos had measurable viral RNA loads in the swab samples after day 21, and continued being positive at low levels until the end of the experiment. In summary, vRos (the virus corresponding to the inferred ancestral sequence at the black node) and CSFV_Roesrath_P2 showed similar characteristics *in vivo* whereas vRos_S1359N_A2668T (which corresponds to the ancestral sequence inferred at the red internal node) displayed an attenuated phenotype. The observation that vRos_S1359N_A2668T replicates faster than vRos in cell culture, but is less virulent *in vivo*, supports the hypothesis that the substitutions S1359N (in NS2) and A2668T (in NS4B) are adaptations specific for cell culture.

## Discussion

In this study, we have investigated individual genotypes and phenotypes in a population of the RNA virus CSFV strain “Roesrath”. Until now no functional cDNA clone has been established from “Roesrath” or other recent genotype 2.3 strains.

In addition to deep sequencing the entire virus population, we created 84 unique full-length cDNA clones, 70 of which were fully sequenced. This unique data set allowed us to perform detailed investigations of the circulating genotypes. We also determined phenotypes of RNA transcripts derived from all cDNA clones. Finally, we reconstructed viral genomes from ancestral sequences corresponding to internal nodes in the virus phylogeny, and investigated their functionality *in vitro* as well as *in vivo* in the natural host system.

The functionality of all 84 cDNA clones was investigated in cell culture. We found that 82% of the investigated cDNA clones were completely non-functional and only 12% were infectious. This finding is consistent with previous results showing a high fraction of lethal and detrimental mutations in offspring of RNA viruses [3], [4], [32], [35]. We note that this may be problematic if the goal is to obtain functional, high-fitness molecular clones for investigations of virulence and pathogenesis. It also suggests that such clones (or sequences obtained from them) may not provide the best basis for understanding the functional form of the virus. However, we also show how computational methods can be used to deduce the sequence of viruses that are functional. We did this by inferring the sequence of ancestors of the circulating forms, since these must have been functional. Importantly, when reverse genetics was used to test the constructs, it was found that the inferred RNA sequences were indeed infectious in cell culture and that the rescued viruses were virulent in pigs. We propose that the approach of using ancestral reconstruction to infer the sequences of functional viruses, followed by construction and analysis of molecular clones corresponding to these ancestors, would be useful for analysis of many other RNA viruses, and that this should allow a better understanding of both their functionality and virulence. We also propose that these ideas may have relevance for bioinformatical analysis of virus sequences. When performing phylogenetic reconstruction of virus sequences, it is generally assumed that a common substitution process has operated in the entire tree. This substitution process is a mix of the effects of mutation and subsequent selection. However, in a viral data set obtained by sequencing individual virus genomes isolated from infected hosts, a majority of sequences are likely to correspond to non-functional variants. These sequences have undergone mutation, but have not been tested by subsequent selection, except for their ability to assemble virus particles. The external branches on such phylogenetic trees therefore correspond to a different substitution process (mostly mutational events) than the internal branches (mutation and selection). It is possible that explicitly accounting for this difference could be useful for phylogenetic analysis of viral sequences.

The population heterogeneity found in the CSFV “Roesrath” isolate is similar to *in vivo* variation found for other genotype 2 medium virulent strains [9]. *In vivo* studies of highly virulent CSFV strains display higher levels of heterogeneity compared to the medium or low virulent strains [9], [15]. It is unknown if such initial heterogeneity is necessary for high virulence or whether it is generated inside the host. We have here shown that virus derived from cDNA clones can be as highly virulent as a wildtype isolate [15]. Functionality of the cDNAs *in vivo* is probably even more restricted compared to *in vitro* because there is added pressure from the host response. A fully functional cDNA clone *in vitro* might turn out to be impaired *in vivo* [15] as is seen for the vRos_S1359N_A2668T.

This study combines reverse genetics and NGS for exploring viral population structure in depth. NGS alone is limited to SNV calling and reconstruction of haplotypes, but the latter is mostly useful in highly heterogeneous populations, where overlaps of SNVs between individual sequencing reads are abundant. We have shown that the additional use of full genome sequencing of cDNA clones for reverse genetics can provide powerful new data, which can be used to determine haplotype structure, explore phenotypes, and identify mutations of interest. We suggest that these approaches with benefit could be applied to other RNA viruses, thereby leading to better understanding of the viral population dynamics of these important pathogens.
